# The methylated way to translation

**DOI:** 10.18632/oncotarget.22073

**Published:** 2017-10-25

**Authors:** Ruiqi Han, Boris Slobodin, Reuven Agami

**Affiliations:** Reuven Agami: Division of Oncogenomics, The Netherlands Cancer Institute, Oncode Institute, Amsterdam, The Netherlands; Department of Genetics, Erasmus University Medical Center, Rotterdam, The Netherlands

**Keywords:** transcription, m^6^A, translation, RNA, modification

Metabolism of messenger RNAs (mRNAs) consists of multiple steps, from transcription, through splicing, export to the cytoplasm, localization, translation to proteins and, finally, degradation. These steps, which are crucial to ensure correct genetic expression, have long been considered as separate events occurring at distinct time points and different locales. Recent studies suggest that they are not only interconnected, but might also be coupled to the initial process - transcription.

**Figure 1:**
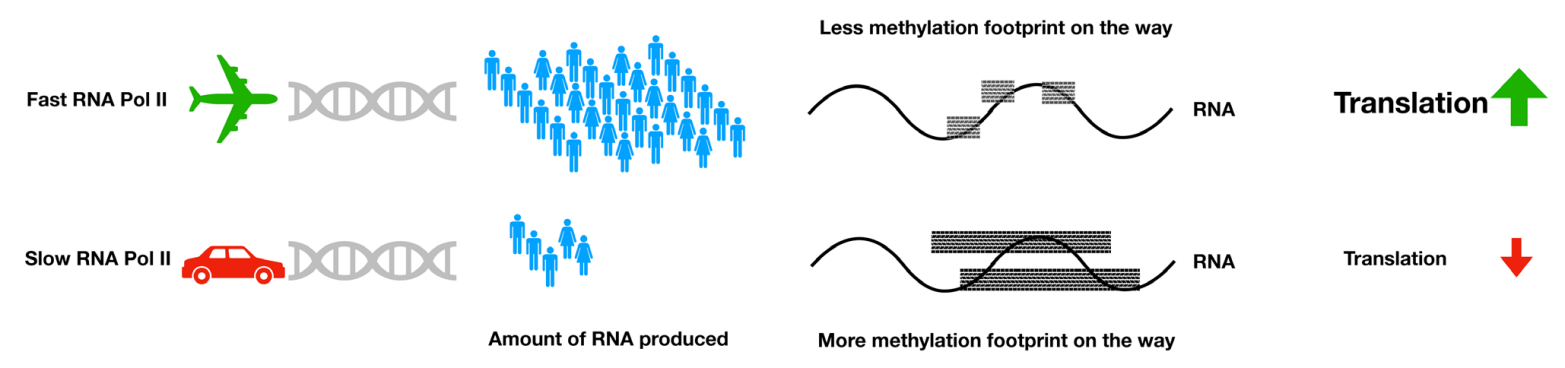
Different transcription rates dispose distinct m^6^A footprint on mRNAs, which profoundly impact translation efficiency.

Initial studies in the field showed that the occurrence of exon inclusion and intron retention could be altered in HEK293 cells with “slow” and “fast” RNA Polymerase II (RNAPII) mutants, suggesting that the rate of transcriptional elongation is crucial for the appropriate splice form [1] (Figure 1). Another important step of mRNA life is its export from the nucleus into the cytoplasm. It has been suggested that the export is not merely a sequential event, but a tightly regulated process that might depend upon transcription. In yeast, a group of factors that function in transcription elongation and mRNA export form a “Transcription/Export” (TREX) complex, which couples to RNA Pol II throughout the entire RNA molecule [2].

As splicing and export could occur co-transcriptionally, they might be directly affected by the dynamics of transcription. However, how post-transcriptional processes - such as degradation and translation - could be coupled to transcription? When a single-molecule mRNA decay study measured stabilities of two mitotic mRNAs, SWI5 and CLB2 in yeast, they found that the precise control of their cytoplasmic decay is largely regulated by their promoters via co-transcriptional binding of a co-factor, Dbf2p [3]. Similarly, Bregman et al. showed that promoter sequences can recruit Rap1p protein to enhance the decay of the transcribed mRNA in yeast [4]. Thus promoters, elements that drive the transcription, are capable of regulating the destiny of mRNAs after its export into the cytoplasm. Recent evidences from several studies suggest that also the mRNA translation could be regulated, to a large extent, by transcription. First, yeast promoters were shown to recruit RNAPII subunits to facilitate translation of specific transcripts [5]. Similarly, upon glucose starvation, specific yeast promoters were shown to bind Hsf1 to direct mRNA localization and the efficiency of translation in the cytoplasm [6]. These two studies demonstrate the ability of yeast promoters, via co-transcriptional recruitment of effector proteins, to regulate translation of specific mRNAs.

Recently, we discovered a direct genome-wide link between transcription and translation in mammalian cells [7]. While testing the effect of human promoters on translation, we identified a positive correlation between the levels of mRNA expression on their capabilities to bind ribosomes (i.e., translational efficiency, or TE). However, we observed that mRNA levels present in the cytoplasm are not the factor that dictates TE directly. Instead, we found that rates of transcription (i.e. the strength of the promoter activity to recruit RNAPII and the speed of transcriptional elongation) positively regulate TE across different mammalian cell lines.

To study how this link is maintained, we examined a possible role of a specific RNA modification, N6-methyladenosine (m^6^A), in coordinating the two processes [7]. Indeed, upon knock-down of several m^6^A-regulatory factors, both “writers” and “readers”, we observed a striking positive effect on the TE of mRNA that was characterized by slow transcription rates. Direct immunoprecipitation of m^6^A-modified mRNAs demonstrated significant difference between the methylation levels of the repressed and induced transcripts, suggesting that mRNAs that are “repressed” (i.e., possess low levels of transcription), have higher m^6^A content. This, in turn, represses TE, therefore linking inefficient transcription to inefficient translation. Finally, by artificial slow-down of RNAPII dynamics, we showed that the process of m^6^A modification on mRNA is likely to be co-transcriptional.

Our study provides an additional evidence that transcription machinery is intrinsically linked with the process of translation and support the theory that the fate of a mRNA molecule could be in part pre-determined during transcription. Future studies should address the hidden link between RNAPII and m^6^A methylation complex and reveal how the speed of RNAPII is translated into the m^6^A content deposited on the transcript. Additionally, the exact role of m^6^A in translation still remains ambiguous. While we proposed an inhibitory role of m^6^A in translation, several recent studies found it stimulatory [e.g., 8]. The paradox could be partially explained by the precise location of the modified residue within the transcript (e.g., coding region versus untranslated regions). Future research should examine this hypothesis and uncover how cells determine the precise sites of m^6^A deposition.

mRNAs are modified by a myriad of epigenetic modifications of which little functional knowledge is present. The functional investigation of the role of these RNA modifications is just emerging, and is expected to increase in volume in the coming few years. With the increasing knowledge of m^6^A regulation, its role in gene expression, involvement in diseases and the potential to serve for diagnosis (e.g. in cancer) is expected to become much clearer in the near future. The possible applications of RNA modifications, including drug development targeting m^6^A machineries and thus altering the epigenetic environment, could open up new avenues to a better and more effective diagnosis and treatment of human diseases stemming from imbalanced gene expression.
